# Short-term impact of sediment addition on plants and invertebrates in a southern California salt marsh

**DOI:** 10.1371/journal.pone.0240597

**Published:** 2020-11-05

**Authors:** Kaelin J. McAtee, Karen M. Thorne, Christine R. Whitcraft

**Affiliations:** 1 Department of Biological Sciences, California State University Long Beach, Long Beach, CA, United States of America; 2 United States Geological Survey (USGS), Western Ecological Research Center, Davis, CA, United States of America; University of Sydney, AUSTRALIA

## Abstract

The implementation and monitoring of management strategies is integral to protect coastal marshes from increased inundation and submergence under sea-level rise. Sediment addition is one such strategy in which sediment is added to marshes to raise relative elevations, decrease tidal inundation, and enhance ecosystem processes. This study looked at the plant and invertebrate community responses over 12 months following a sediment addition project on a salt marsh located in an urbanized estuary in southern California, USA. This salt marsh is experiencing local subsidence, is sediment-limited from landscape modifications, has resident protected species, and is at-risk of submergence from sea-level rise. Abiotic measurements, invertebrate cores, and plant parameters were analyzed before and after sediment application in a before-after-control-impact (BACI) design. Immediately following the sediment application, plant cover and invertebrate abundance decreased significantly, with smothering of existing vegetation communities without regrowth, presumably creating resulting harsh abiotic conditions. At six months after the sediment application treatment, *Salicornia bigelovii* minimally colonized the sediment application area, and *Spartina foliosa* spread vegetatively from the edges of the marsh; however, at 12 months following sediment application overall plant recovery was still minimal. Community composition of infaunal invertebrates shifted from a dominance of marsh-associated groups like oligochaetes and polychaetes to more terrestrial and more mobile dispersers like insect larvae. In contrast to other studies, such as those with high organic deposition, that showed vegetation and invertebrate community recovery within one year of sediment application, our results indicated a much slower recovery following a sediment addition of 32 cm which resulted in a supratidal elevation with an average of 1.62 m (NAVD88) at our sampling locations. Our results indicate that the site did not recover after one year and that recovery may take longer which illustrates the importance of long-term monitoring to fully understand restoration trajectories and inform adaptive management. Testing and monitoring sea-level rise adaptation strategies like sediment addition for salt marshes is important to prevent the loss of important coastal ecosystems.

## Introduction

Coastal wetlands, including salt marshes, are among the most productive ecosystems in the world and are recognized for their ecological functions including habitat provision, water filtration, flood abatement, and fishery and biodiversity support [1,2]. Despite their importance, over half of global wetlands have already been lost as a result of human activities [2,3], and remaining coastal wetlands are threatened by climate change, specifically sea-level rise (SLR) [4]. Under moderate SLR scenarios, up to 59% of the remaining global coastal wetlands could be lost by 2100 [5].

Under conditions of sufficient sediment supply and accommodation space, feedbacks between biological and physical processes control accretion, allowing the salt marshes to maintain their elevation relative to tidal inundation and increasing SLR rates [6–13]. However, today, many coastal salt marshes are unable to build their elevations enough to outpace accelerated rates of SLR. This is especially key for those marshes that are located in modified landscapes that are experiencing subsidence and/or alterations of sediment dynamics, such as decoupling rivers from estuarine systems and river channelization [14,15]. Additionally, many intensely urbanized estuaries are surrounded by developed land that limits the ability of salt marshes to migrate landward [16]. For these marshes, management strategies have been developed to minimize their loss due to SLR.

One such strategy to artificially increase salt marsh elevations is sediment addition, a process that mechanically adds a layer of sediment to increase surface elevations, reducing overall inundation and often increasing primary productivity [17]. Impacts and results of sediment addition have been studied in other regions of the world as well as marshes on the East and Gulf Coasts of the United States [18–25]. Many of these sediment application studies have been conducted in microtidal deltaic salt marsh systems where there is some available natural sediment supply. Fewer studies have been conducted in urbanized, sediment-limited salt marsh ecosystems. To date, only a few limited studies have analyzed a thin-layer sediment addition in salt marshes on the Pacific Coast of the United States [26,27], making this research timely. Our study highlights the plant and invertebrate community change under a deeper sediment application project with an elevation goal designed to offset high SLR risk and local subsidence.

For coastlines experiencing higher relative SLR rates, there is a sense of urgency to develop effective adaptation strategies to prevent salt marsh loss. Coastal salt marshes have been projected to become submerged over the coming century often due to limited sediment supply or landscape modifications that prohibit upland migration [13,15,28–30]. These at-risk ecosystems provide an opportunity to implement and test SLR adaptation strategies to prevent loss of these ecosystems. Thus, additional studies are needed to assess the effects of large-scale sediment addition projects that deposit thicker sediments to offset elevation deficits in locations that are experiencing higher SLR rates.

Monitoring and studies of sediment application projects often focus on elevation change and vegetation but do not monitor other key ecosystem components including the invertebrate communities. Yet, the abundance, diversity, and community structure of invertebrate communities (polychaetes, oligochaetes, amphipods etc.) can serve as indicators for marsh health [31]. Earlier studies on subsiding coastal marshes in Louisiana [20,32] found that moderate levels of sediment addition (12–14 cm) could allow for the macro-invertebrate community to recover to pre-sediment application conditions; however, too much sediment (~17 cm) could impair recovery. Despite the recognized importance of invertebrates, few studies have experimentally studied the short-term responses of belowground invertebrates, including micro-invertebrates, to plant disturbance and thin-layer sediment addition.

Given that other studies had seen recovery of plants within a one to two-year time frame (as summarized in Raposa et al. 2020 [33]), the objectives of this study were to monitor the shorter-term effects (within 12 months) of large scale deeper sediment addition project on (1) the vegetation community (percent cover, community composition), (2) the benthic invertebrate community (abundance, species richness, diversity and community composition) and (3) associated abiotic parameters (elevation, porewater salinity, temperature and light intensity). We hypothesized that all plant and invertebrate parameters would decrease following sediment application. However, we predicted that the plant community would recover but on a longer time scale as seen in non-sediment application restoration studies and that this would facilitate recovery of invertebrate communities.

## Materials and methods

### Study site

**Fig 1:**
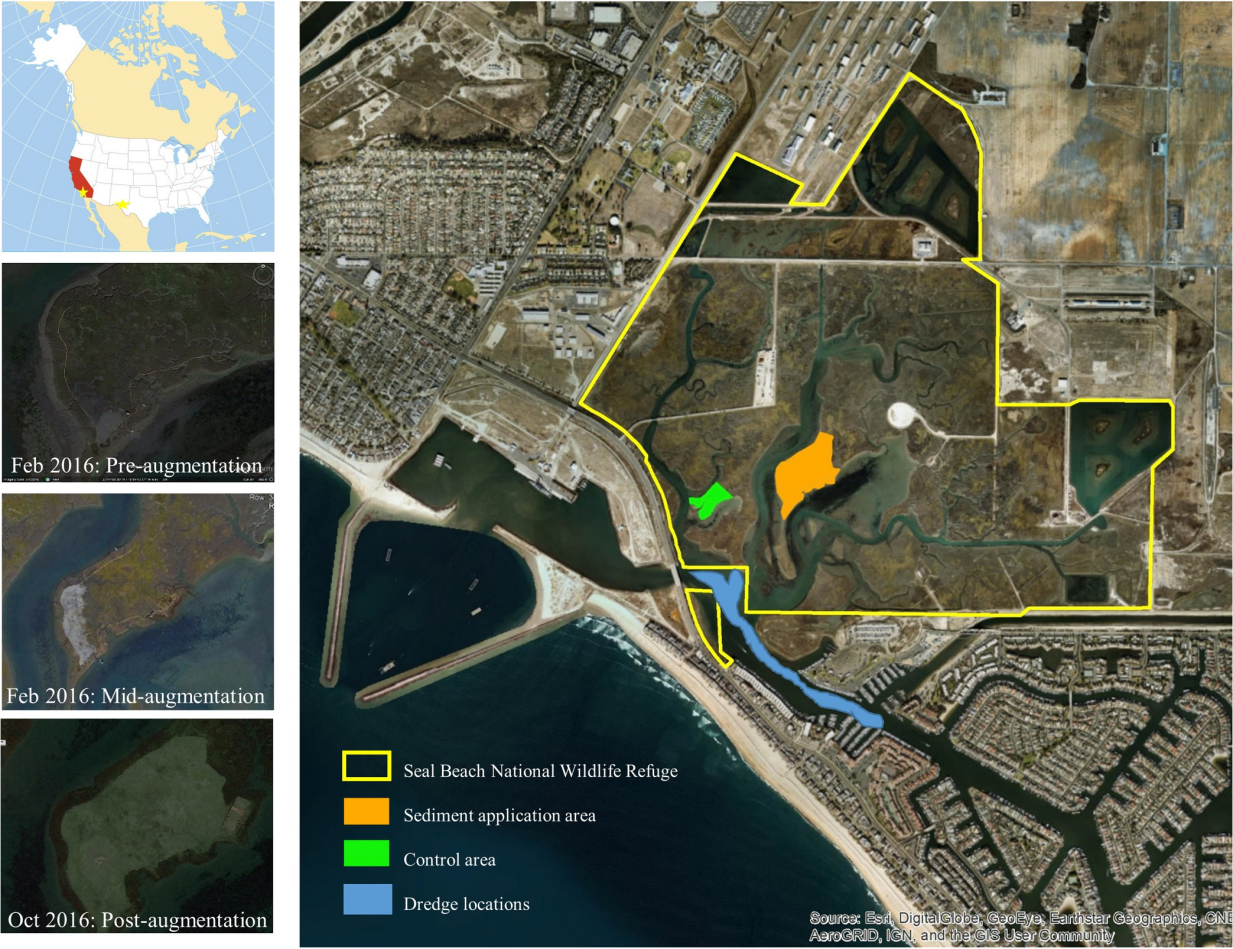
Control and sediment application sites within the Seal Beach National Wildlife Refuge shown in relation to the dredge locations in neighboring Anaheim Bay. Aerial images show the addition of sediment through time from early February 2016 (pre-sediment application) through end of February 2016 (midway through sediment application) until October 2016 (post-sediment application). Image data: Google, Maxar Technologies.

This study was conducted at the Seal Beach National Wildlife Refuge (SBNWR) located in Orange County, California, USA on the Naval Weapons Station, Seal Beach (33° 44' 17.99" N, -118° 03' 60.00" W) (Fig 1). SBNWR is managed by the U.S. Fish and Wildlife Service (USFWS) and comprises 965 acres, with 750 acres of tidal marsh and three intertidal and sub-tidal restored ponds [28,34]. This marsh is primarily dominated by *Spartina foliosa* (Pacific cordgrass) and *Batis maritima* (saltwort) as well as an invertebrate community dominated by oligochaetes and polychaetes. SBNWR is also home to migratory birds and protected species including California Least Terns (*Sternula anntillarum browni*) and East Pacific green sea turtles (*Chelonia mydas agassizii*). SBNWR includes one of the largest populations of light-footed Ridgway’s rail (*Rallus longirostris levipes*) in California. The Ridgway’s rail uses *S*. *foliosa* to build nests in the marsh [35]. At high tides in the SBNWR, *S*. *foliosa* is almost completely submerged in water, which drastically minimizes nesting habitat for the Ridgway’s rail and was one of the observations that initially motivated SBNWR managers to initiate studies of marsh elevation. To conduct this research, special use research permits were obtained from USFWS and the US Navy; collection permits were obtained from California Department of Fish and Wildlife.

The SBNWR located in southern California provides an ideal setting to assess the effects of sediment addition using a thicker sediment application process to prevent SLR submergence. This salt marsh has been identified as being at a higher risk of submergence due to accelerated rates of relative SLR, lower rates of accretion due to the estuary being sediment limited, and surrounding urban development that prevents landward migration [28,36]. The current rate of SLR in southern California is 2.1mm/yr [37], and when combined with local subsidence rates, the SBNWR is experiencing a relative SLR rate of 6.23 mm/year [36].

Management strategies to offset the threat of SLR in SBNWR were considered and included restoring natural freshwater inputs and sediment supply, decrease regional subsidence rates, and removing infrastructure to allow for landward migration; however, these options were unrealistic for SBNWR in the near-term. Therefore, a near-term feasible approach was determined to be sediment addition, and this site is a useful case study for similar urbanized marshes around the world.

### Sediment application methodology

The sediment application methodology was designed and implemented by the USFWS and various researchers from a range of agencies and universities. Dredging was contracted by Orange County Public Works and was dredged from an adjacent subtidal area near SBNWR in Anaheim Bay (Fig 1). The project goals were to spread a fairly uniform thin layer of sediment and maintain a minimum 7.62 cm increase in the marsh plain elevation within the project site two years after sediment addition [27]. Using sediment spray equipment sediment application began in late January 2016 and ended in early April 2016 over a 4.05-hectare area for 31,094 m^2^ 901 of dredge material [27]; depth of applied sediment varied across the site with an average of 25.4 cm [27].

Due to challenges in obtaining the sediment material from Anaheim Bay, the dredged sediment grain size was sandier and contained lower organic matter than the original soils at SBNWR when compared to the control site (pers. comm. K. Gilligan). Hay bales, straw wattles, sand bags, and geotextile fabric were placed along the perimeter of the sediment application site to minimize loss of sediment to nearby channels during the application process. While suspended sediment concentrations did increase immediately adjacent to the application area, these engineering interventions were successful in retaining sediment overall [27]. The goal of the USFWS for this sediment application project was intended to build elevation capital relative to SLR and enhance marsh plain elevations for *Spartina foliosa*, Ridgway’s Rail primary habitat requirement.

### Sampling design

Pre-sediment application plant and invertebrate data were collected in the spring and fall of 2015 (referred to as PRE-spring and PRE-fall). The first post-sediment application sampling period occurred in May 2016, one month after treatment (1 MAT). Subsequent samples were taken six months after treatment (6 MAT) in the fall of 2016 and 12 months after treatment (12 MAT) in the spring of 2017. Two areas of the refuge were used for the study: the experimental sediment application site (experimental site or Exp.) which received the sediment slurry, and a control site which is located across the channel from the sediment application site and did not receive the sediment slurry (Fig 1). Pre-sediment application, both sites had statistically similar total plant cover and species richness (p > 0.05 all tests). The vegetation community composition was statistically similar between the two areas and was classified by elevation and dominant plant type into habitat types. Both sites had low marsh areas that were either *Spartina foliosa*-dominated or ponded areas with permanent standing water as well as middle to high marsh areas that were *Batis maritima-*dominated. In addition, the control and sediment application site had similar elevations and experienced similar sedimentation patterns and tidal inundation [34].

Sampling took place over five time points (PRE-fall, PRE-spring, 1 MAT, 6 MAT, 12 MAT) in three dominant habitat types: (1) *Spartina foliosa*-dominated (Spfo), (2) *Batis maritima-*dominated (Bama), and (3) ponds or standing water (Pond). To be placed in a habitat category, an area had to contain >50% cover of that vegetation type. We included the three habitat types because we expected each habitat to respond in different ways given their different starting states. Random points were generated for both the control and sediment application site (50 points in the sediment application site and 20 points in the control site) using the random point generator in ArcView, ensuring that no points landed in the 1m buffer zone surrounding the sediment application area. To choose sampling sites, each random point was visited, and habitat type was determined based on a qualitative assessment of the dominant plant community. This process was repeated for subsequent points until eight locations in each particular habitat type were identified (eight locations per habitat for a total of 24 locations in the sediment application site). Once this had been established, plant and invertebrate sampling took place (see methods below). A similar protocol was followed for sampling the control site, with a total of five locations per habitat (for a total of 15 locations) due to the smaller area. For post-sediment application sampling, the same sites sampled pre-sediment application were studied, and changes in habitat type were noted. The control site is smaller in area than the experimental sediment application site due to constraints on space availability in SBNWR (Fig 1). These constraints included choosing a control site that had similar marsh elevation to the sediment application site, was not in the active shooting range of the Naval Weapons Station and did not contain Light-footed Ridgeway rail nesting sites.

### Abiotic parameters

To determine pre-sediment application elevation of sampling locations we used a digital elevation model (DEM) raster from Buffington et al. 2018 [38]. Post-sediment application elevations were determined using a DEM from a photogrammetry survey on April 18, 2016 [39]. Using ArcMap 10.5, we extracted pre- and post-sediment application elevations by sampling point. The difference between pre and post-sediment application was then calculated for the sediment addition area. We did not calculate an elevation difference in the control site since any elevation change would be within the error of the DEMs.

USGS collected absolute inundation data from within channels surrounding the sediment application and control sites, measuring levels in North American Vertical Datum of 1988 (NAVD88) [39]. Relative water level data were calibrated to absolute water levels via linear regression in Matlab 2014a [39]. These data were used to calculate the percent of time that the sediment application and control sites were inundated [39], and those values are reported and interpreted in this study.

Porewater soil salinity of the top 0.5 cm (practical salinity units [psu]) was measured at each plot for each sampling season by squeezing porewater from the sediment surface through a Whatman number 1 qualitative grade filter onto a hand-held salinity refractometer. Temperature and light intensity at the sediment surface were measured using loggers (Onset HOBO Pendant Temperature/Light Data Logger) that were placed at five plots in the sediment application site and 5 plots in the control site. These loggers collected continuous data for six weeks following sediment addition. Sediments cores (4.8 cm diameter x 2 cm) were collected from haphazardly chosen sites on both the control and sediment application sites in fall 2015 (pre-sediment application) and in spring 2016 (during and post-sediment application) for analysis of particle size via the hydrometer method [40] and for organic matter content using loss on ignition methods [41].

### Plant community

Total plant cover was estimated for the entire plot using species-specific visual estimations as a specific percent cover within 0.125m^2^ PVC quadrats. Species richness, evenness, and diversity (S, J’, H’) were calculated from these data for each sampling point. Although sampling sites are named after the dominant plant type, some *S*. *foliosa* was present at all sites.

### Invertebrate community

Epifauna visible on the sediment surface (primarily *Cerithideopsis californica* [California horn snail], *Melampus olivaceus* [salt marsh snail], *Geukensia demissa* [ribbed mussel]) were counted within the same quadrat used for plant identification (0.125m^2^). Infauna (e.g., polychaetes and amphipods) were collected using a 4.8 cm diameter, 18.1 cm^2^, corer inserted to a depth of 2 cm [42,43]. We selected a 4.8 cm diameter core to target macrofauna typically in the 1–2 mm size range, recognizing that this is likely to exclude megafauna, such as large clams or crabs. This core size is consistent with published literature on macrobenthos from this and nearby marshes [44,45]. Most (78–89%) of the macrofauna in southern California *Spartina foliosa* marshes is found in the top 2 cm of sediment and is therefore the focus of this study [44]. Cores were transported back to the lab and preserved in 8% buffered formalin and stained with Rose Bengal. The sediment samples were sieved through a 300 μm mesh sieve. Invertebrates retained on the sieve were then identified down to the lowest taxonomic classification possible and were stored in 70% ethanol as vouchers. Abundance of benthic infauna, species richness, evenness, and diversity (S, J’, H’) were calculated for each plot.

### Statistical analyses

This study used a BACI design (Before-After, Control-Impact), which is useful in detecting nonrandom change in a series of observations made before and after manipulation of a single system [46]. A body of research by Underwood [47,48] describes the limits of the BACI design when multiple control sites are not available; however, multiple controls are not often available as in this project. Stewart-Oaten and Bence 2001 [46] found that the BACI design is sound for use with careful statistical analyses and detailed interpretation of monitored parameters and results. The experimental design of this study was constrained by available space for replication within SBNWR areas (Fig 1).

The BACI design is preferred over a simple Before-After comparison within the sediment application site because it takes into account the temporal change that may have occurred in the absence of the impact (any changes accounted for in the control site). This allows for changes over time in the impact area that may be unrelated to the impact itself to be controlled for by detailing changes over time in the control area. The differential change, or the BACI effect, is evidence of the environmental impact. In this study, the BACI effect (or significant interaction term, Eq 1, μ = mean) is demonstration of an impact from thin-layer sediment addition as compared to a control site within the same marsh that is unaffected by sediment application.

$$\left(BACI\;effect=(\mu_{Experimental}Before\;-\mu_{Experimental}After)\;-\;(\mu_{Control}Before\;-\mu_{Control}After\right)$$ Eq 1

This BACI effect represents the potential impact of sediment application itself and minimizes the potential impact that temporal effects and any site effects may have on the data.

Using R statistical software and the script written by Schwarz 2015, the BACI effect was estimated for all parameters [49]. A two-way ANOVA (or permutational ANOVA, when assumptions of an ANOVA were not met, p(mc) is the p-value drawn from Monte Carlo samplings) was used to test for significance of the BACI effect (Y = SiteClass Period SiteClass*Period), where SiteClass represents the effect of the Control vs. Impact sites, Period represents the before vs. after contrast, and the SiteClass*Period represents the BACI effect. All results analyzed using the BACI design will be described using the phrase “after controlling for changes that occurred in the control site.” A two-way ANOVA was used to compare temperature and light intensity by SiteClass. In these analyses, date was treated as a random factor since the effect of individual days on these parameters was not of interest.

Multivariate statistics calculated using the Primer statistical software were used to analyze both plant and invertebrate community composition among habitat type and treatment timing. The Bray-Curtis dissimilarity fourth root transformed was used to analyze the similarities and dissimilarities in species composition between pre- and post-sediment application communities through PERMANOVA. Canonical Analysis of Principle Coordinates (CAP) analyses were run on vegetation and infauna invertebrate community composition to visualize differences between samples and determine the strength of the associations (canonical correlations, δ).

Analysis of data demonstrated a strong interaction between treatment Period*Season*Habitat type which was mainly driven by seasonal differences. Relevant literature also documents strong seasonal patterns in vegetation and invertebrate communities in coastal salt marshes [50]. Thus, all data were analyzed within season. The level of significance used in this study is α = 0.05 unless otherwise noted. One standard error about the mean is presented for all data unless otherwise noted.

## Results

### Abiotic parameters

In the sediment application area, the pre-sediment application mean elevation was 1.30 m (NAVD88) with a min of 1.18 m and a max of 1.42 m (NAVD88). The 2016 post-sediment application mean elevation was 1.62 m (NAVD88) with a min of 1.05 m (NAVD88) and max 1.92 m (NAVD88) at sampling locations (S1 Table). The change in elevation between pre and post-sediment application had an average change of 0.32 m (S1 Table). The control site mean elevation was 1.27 (NAVD88, m) with a min of 1.19 m and max of 1.36 m (NAVD88, S2 Table). Greater details of the post-sediment application elevation survey methods are available at Thorne et al. (2017) [39].

**Fig 2:**
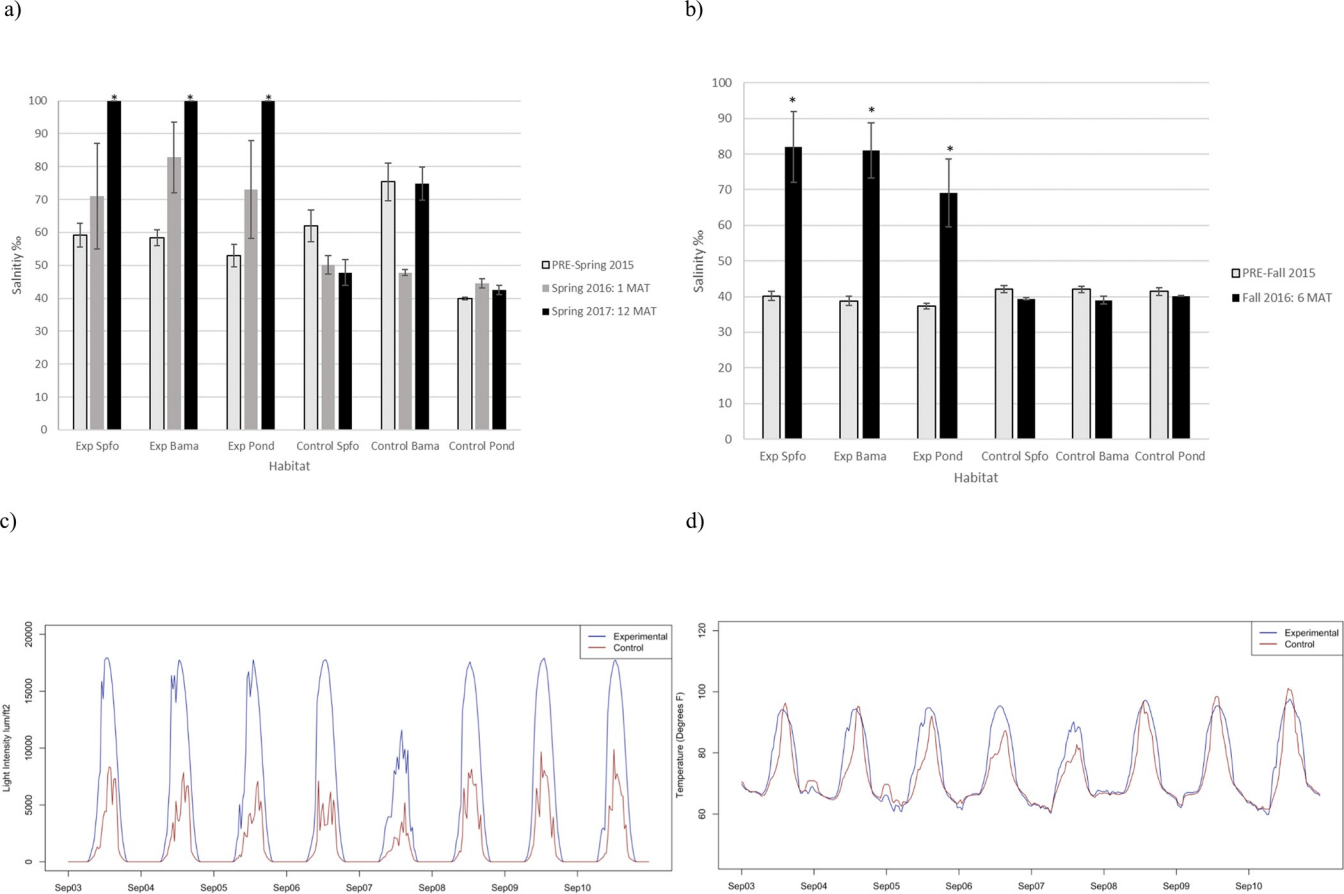
a) Salinity before sediment application (PRE-spring and PRE-fall) and b) after sediment application (1 MAT, 6 MAT, 12 MAT) *indicates a significant change from pre-sediment application levels compared to changes that occurred in the control site. P-values found in S1 Table c) One-week snapshot of light intensity and d) temperature at the control and experimental sites post-sediment application. This one week was selected from a six-week dataset. Habitats are abbreviated as follows: *Spartina foliosa*-dominated (Spfo), *Batis maritima-*dominated (Bama), and ponds or standing water (Pond). Exp is the experimental sediment application area/site. MAT is months after treatment.

Immediately following sediment application (1 MAT), salinity increased in all habitats but was only significantly higher in Bama habitats when compared to changes that occurred in the control site (Fig 2A and S3 Table). Salinity was significantly higher for all habitats at 6 and 12 MAT as compared to pre-sediment application (Fig 2 and S3 Table). Over the six-week period of measurement (summer), temperature (p = <0.001, F = 90.25) and light intensity (p<0.001, F = 114.69) were significantly higher in the sediment application site compared to the control site (Fig 2B).

**Fig 3:**
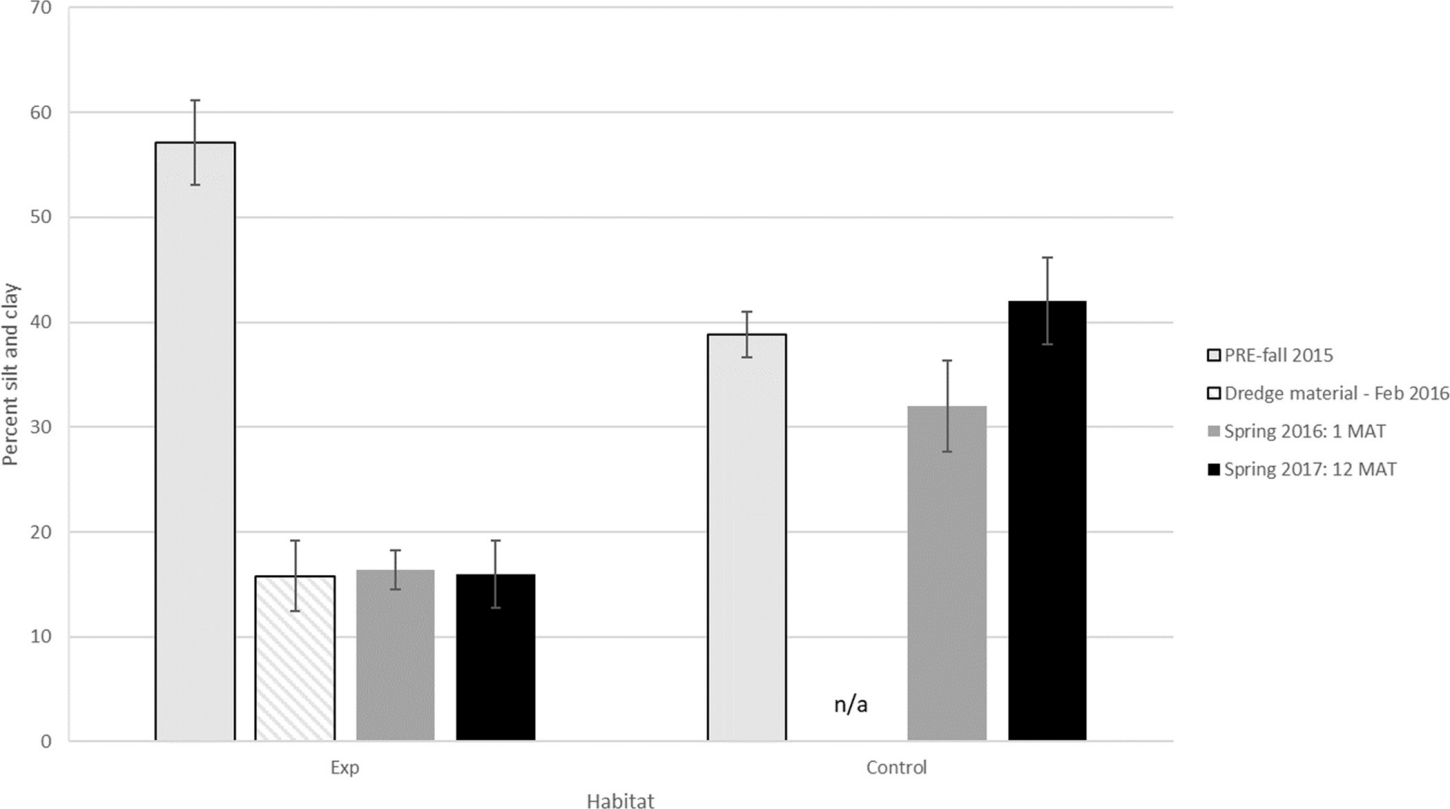
Percent silt and clay in sediment cores collected through time from the control and experimental sites at SBNWR to document key changes. Data are presented from before sediment application (PRE-fall), during sediment application (February 2016) and after sediment application (1 MAT, 12 MAT12MAT).

Due to challenges in obtaining the sediment material, the grain size of the dredge material contained much less silt and clay (15%) than the pre-sediment application grain size or the control site (57%, 38% respectively) (Fig 3). Unlike original marsh sediment grain size and reflecting the spray content, the sediment on the experimental site after sediment application was low in silt and clay content (16%) and low in organic matter (OM) (1.7%) at 1 MAT and 12 MAT (Fig 3) while the control site was statistically equivalent to pre-application values.

### Plant community

**Fig 4:**
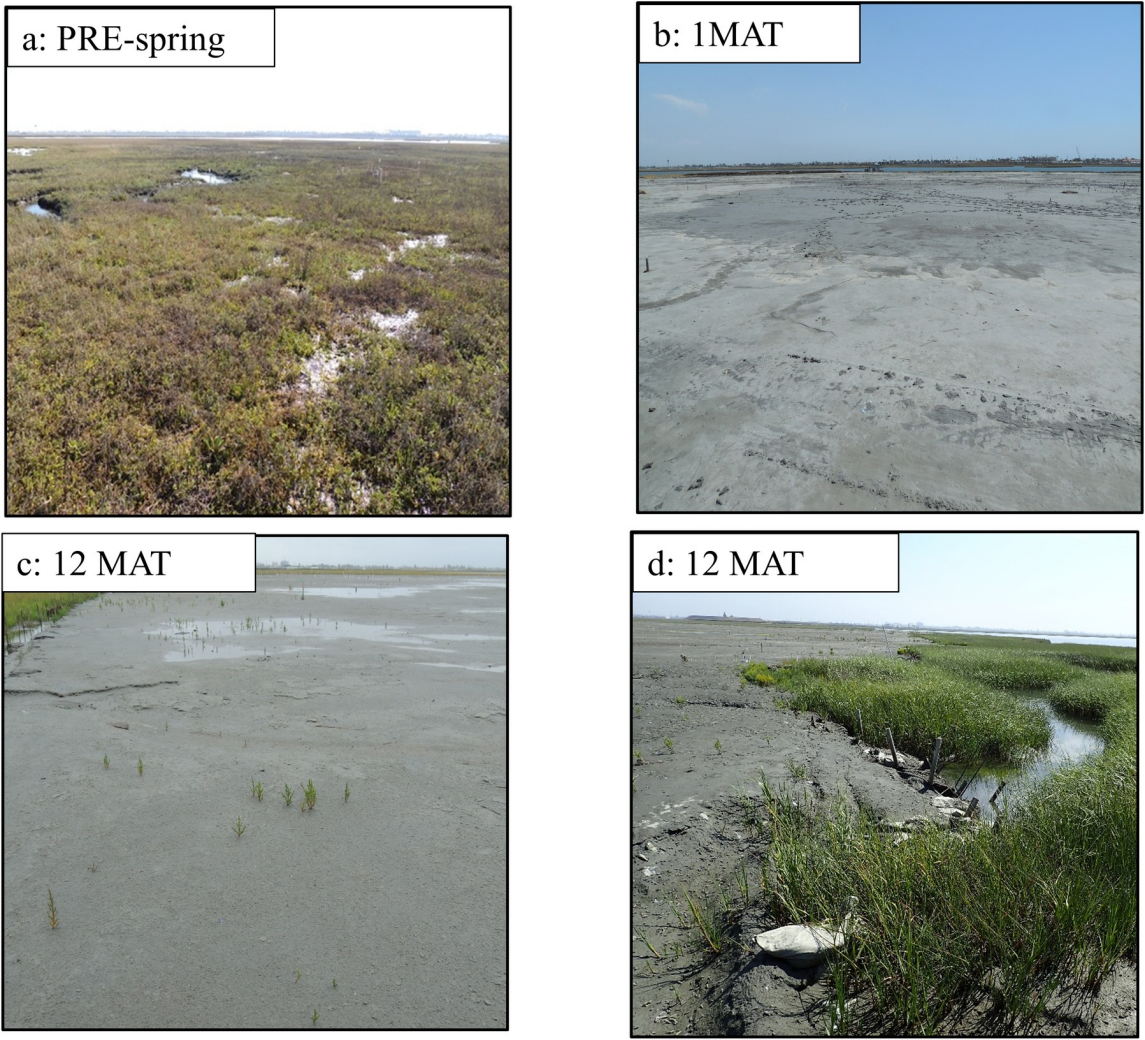
Experimental site photos through time taken from the same location at northeast end of the experimental site. a) Marsh vegetation before sediment application (PRE-spring), b) marsh at 1 MAT, c) *Salicornia bigelovii* beginning to grow in sediment application area at 12 MAT, d) *Spartina foliosa* growing via vegetative spread from vegetated buffer zone (12 MAT). MAT is months after treatment.

**Fig 5:**
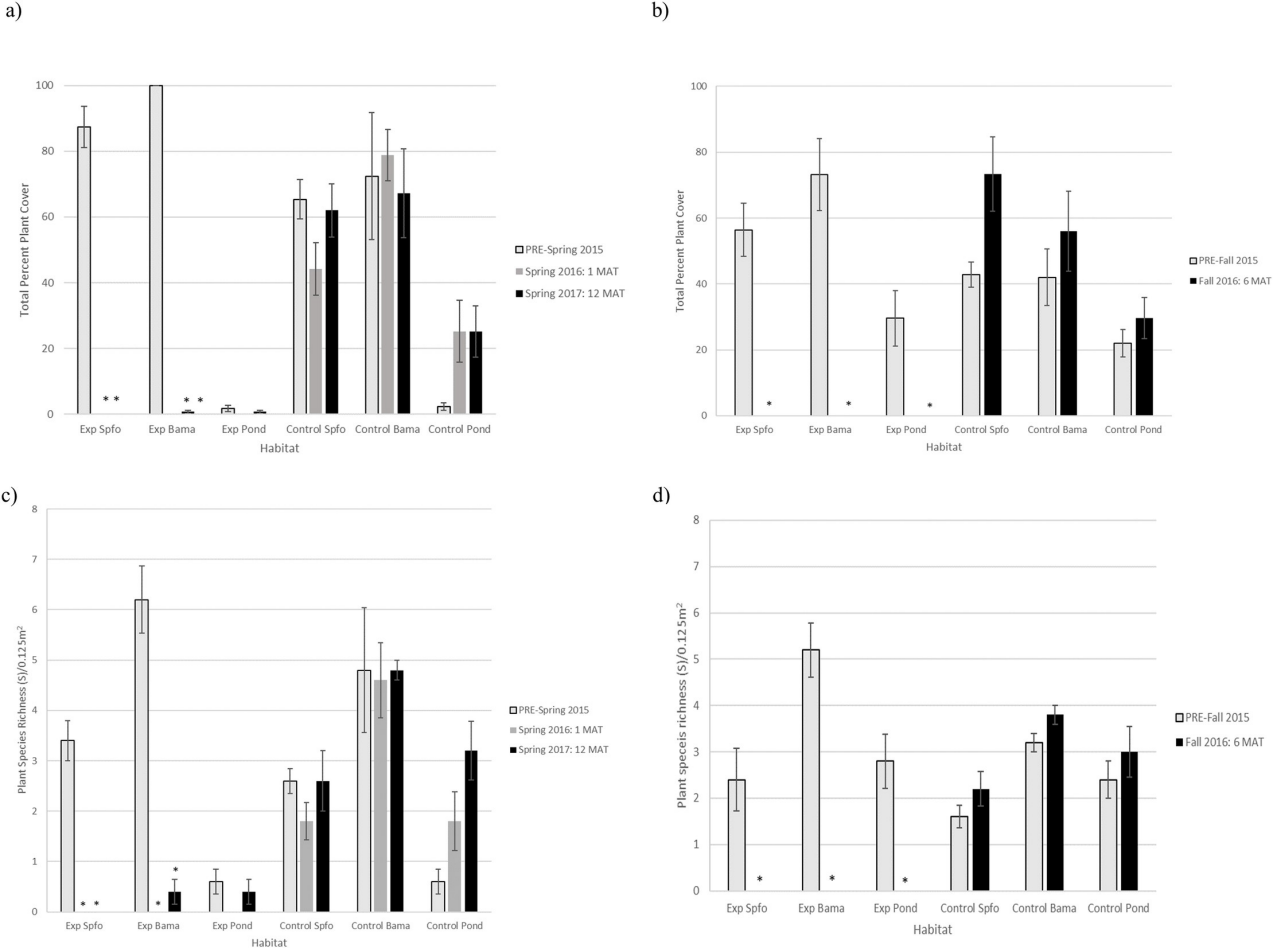
Total plant cover in a) PRE-spring compared to 1 MAT, 12 MAT and b) PRE-fall as compared to 6 MAT. Species richness c) PRE-spring compared to 1 MAT, 12 MAT and d) PRE-fall as compared to 6 MAT. *indicates a significant change from pre-sediment application levels compared to changes that occurred in the control site. P-values found in S4–S6 Tables. Habitats are abbreviated as follows: *Spartina foliosa*-dominated (Spfo), *Batis maritima-*dominated (Bama), and ponds or standing water (Pond). Exp is the experimental sediment application area/site. MAT is months after treatment.

Plants were smothered by the addition of 25 cm of sediment, and no plants were present on the site 1 MAT (Fig 4B). At 6 MAT, *Salicornia bigelovii* (dwarf saltwort) was found growing throughout the site; however, none of these plants fell in our quadrat sites and are therefore not reflected in the data (Fig 4C). Sediment application significantly decreased plant total cover, species richness, evenness, and diversity from pre-sediment application levels for Spfo and Bama habitats 1 MAT and 12 MAT, and for all habitats 6 MAT after controlling for the changes that occurred in the control site (Fig 5 and S4–S6 Tables). Few significant changes occurred between pre- and post-sediment application in the Pond habitat, which remained at low percent cover and species richness both all sampling points (Fig 5 and S4–S6 Tables). At 6 and 12 MAT, plant community composition was significantly different from pre-sediment application due to the absence of plants post-sediment application in all habitats on the sediment application site (S4–S6 Tables).

We observed outside of our quadrat sampling sites that at 12 MAT, *Spartina foliosa* was growing on the edges of the sediment application site via vegetative spread from the surrounding marsh that did not have sediment applied, and *Salicornia bigelovii* was established throughout the marsh, however at very low abundances.

### Invertebrate community

**Fig 6:**
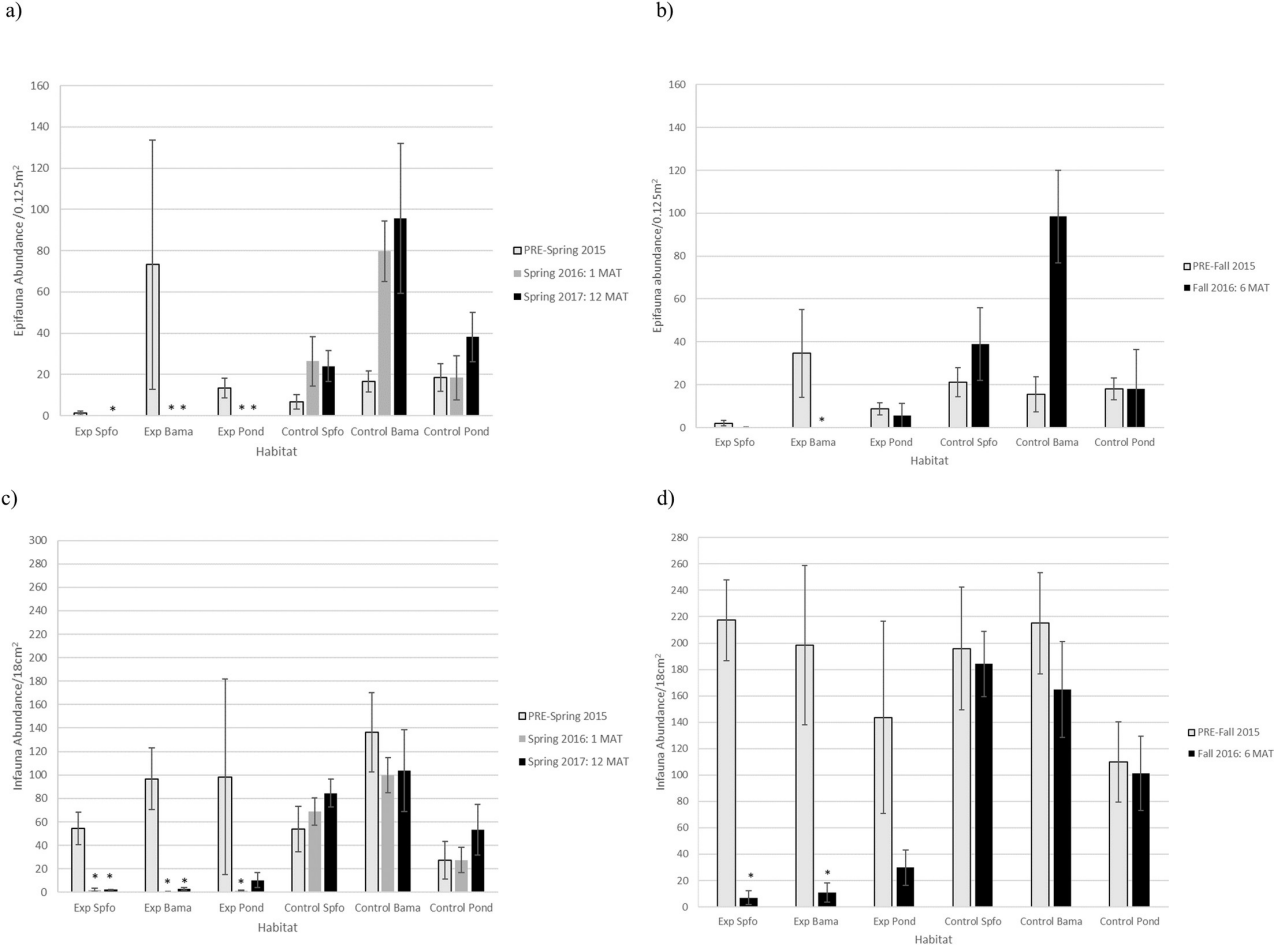
Epifauna invertebrate abundance a) PRE-spring compared to 1 MAT, 12 MAT and b) PRE-fall as compared to 6 MAT. Infauna invertebrate abundance c) PRE-spring compared to 1 MAT, 12 MAT and d) PRE-fall as compared to 6 MAT. *indicates a significant change from pre-sediment application levels compared to changes that occurred in the control site. P-values found in S7–S10 Tables. Habitats are abbreviated as follows: *Spartina foliosa*-dominated (Spfo), *Batis maritima-*dominated (Bama), and ponds or standing water (Pond). Exp is the experimental sediment application area/site. MAT is months after treatment.

Total epifauna abundance (4 species summed) was significantly lower immediately following sediment application (1 MAT) (S7 Table) compared to pre-sediment application abundance in the spring and after controlling for changes that occurred in the control site (Fig 6). At 6 MAT, epifauna abundance was only significantly lower for Bama habitats compared to pre-sediment application after considering the natural variation that happened in the control site (Fig 6 and S7 Table). At 12 MAT, epifauna abundance was significantly lower for all habitat types compared to pre-sediment application levels in the spring and after controlling for changes that occurred in the control site (Fig 6 and S7 Table).

Infauna abundance decreased significantly from pre-sediment application abundance in the spring in all habitat types immediately following sediment application compared to the changes that occurred in the control site (1 MAT) (Fig 6 and S8 Table). At 6 MAT and 12 MAT, infauna abundance increased in all habitat types compared to 1 MAT but was still significantly lower for Spfo and Bama habitats compared to pre-sediment application abundances and after controlling for the changes that occurred in the control site (Fig 6 and S9 and S10 Tables). Species richness, diversity, and evenness decreased significantly in all habitat types immediately following sediment application compared to pre-sediment application levels in the spring and after natural changes were controlled for (1 MAT) (S8 Table). At 6 MAT, richness, diversity, and evenness were not significantly different for Bama and Pond habitats compared to pre-sediment application levels in the fall but were still found to be significantly lower for Spfo habitats after considering the natural changes that occurred in the control site (S9 Table). At 12 MAT, species richness and evenness were significantly lower for Bama habitats, and diversity was significantly lower for Spfo and Bama habitats compared to pre-sediment application levels and after controlling for changes in the control site (S10 Table).

Community composition of infaunal invertebrates was significantly altered following sediment application in all habitat types within the sediment application area relative to the control (S8–S10 Tables). Before sediment application, the infaunal invertebrate community was dominated by oligochaetes and polychaetes, with insects and amphipods comprising a very small percentage of the community. Immediately following sediment application (1 MAT), insects dominated the invertebrate community in Bama and Pond habitats in the sediment application site with a very small representation of oligochaetes and polychaetes. Despite low abundances (discussed above), Spfo habitats 1 MAT retained high percent composition of oligochaetes and polychaetes. At 6 MAT and 12 MAT, all habitat types in the sediment application site were dominated by insects (S8–S10 Tables).

**Fig 7:**
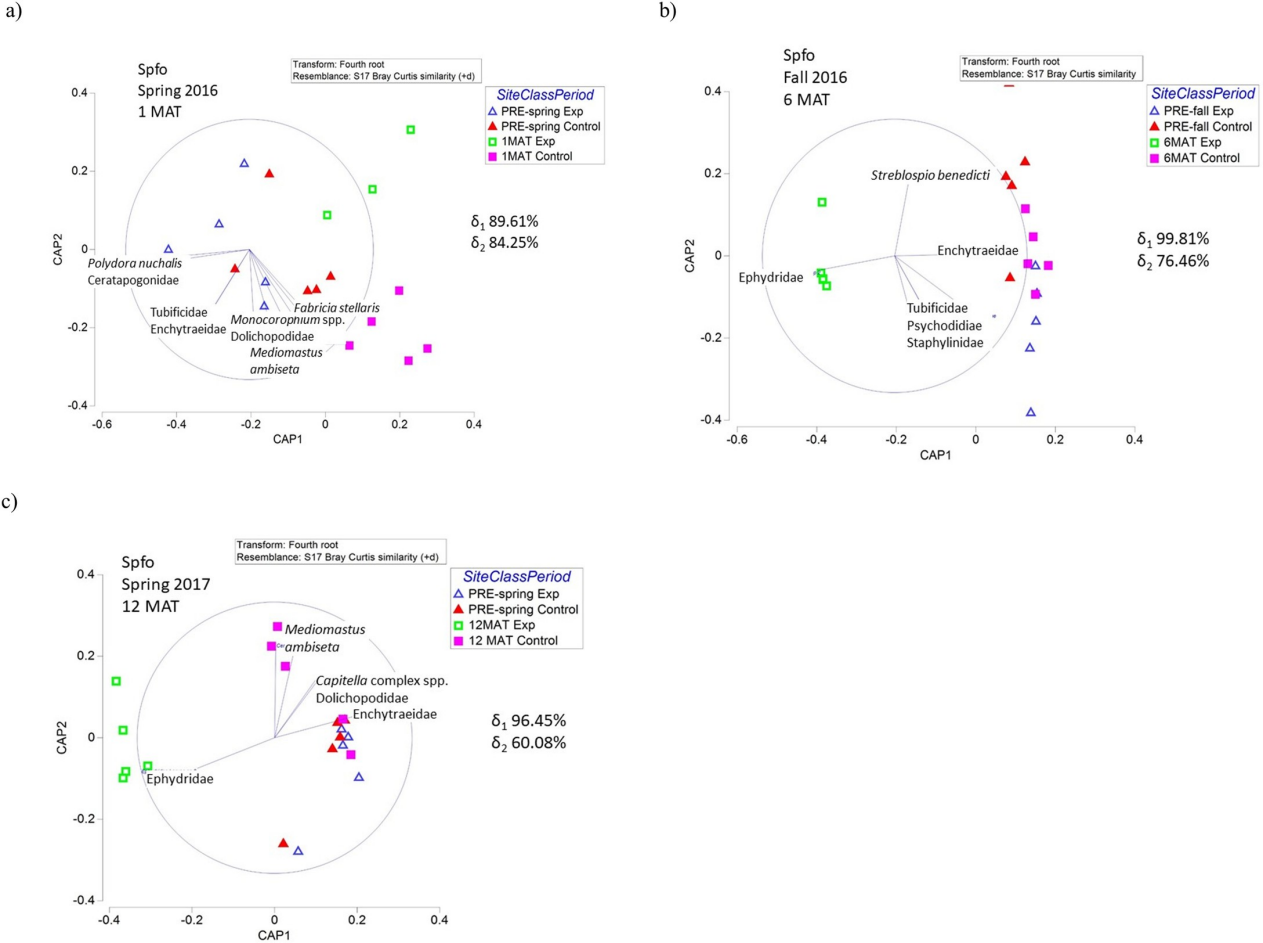
Infaunal invertebrate CAP plots showing the change in community composition of invertebrates for each sampling season (a,b,c) in Spfo habitats compared to pre-sediment application data. Canonical correlation values (δ) for each axis are reported to show strength of the association between the multivariate data cloud and the hypothesized group differences. Habitats are abbreviated as follows: *Spartina foliosa*-dominated (Spfo), *Batis maritima-*dominated (Bama), and ponds or standing water (Pond). Exp is the experimental sediment application area/site. MAT is months after treatment.

**Fig 8:**
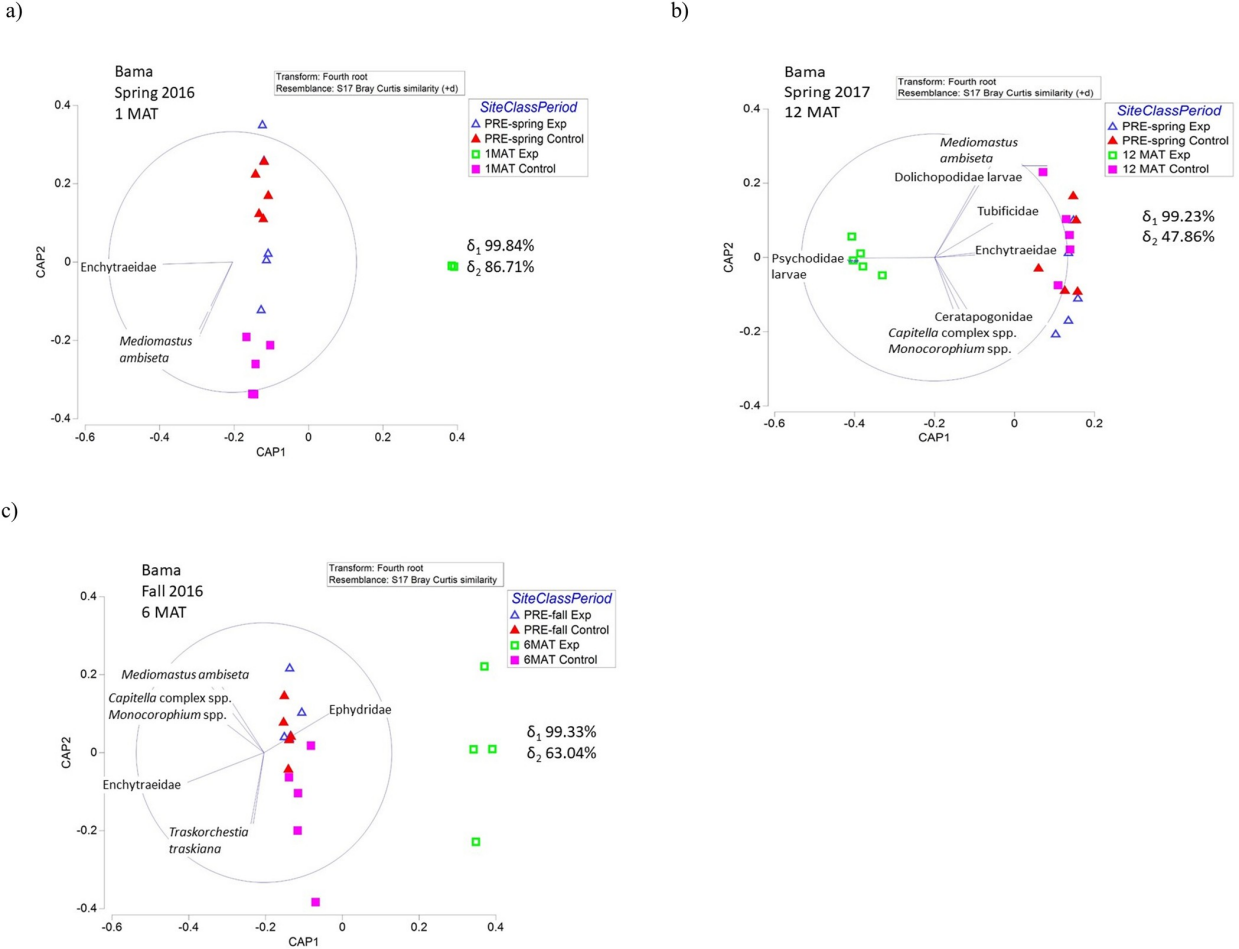
Infaunal invertebrate CAP plots showing the change in community composition of invertebrates for each sampling season (a,b,c) in Bama habitats compared to pre-sediment application data. Canonical correlation values (δ) for each axis are reported to show strength of the association between the multivariate data cloud and the hypothesized group differences. Habitats are abbreviated as follows: *Spartina foliosa*-dominated (Spfo), *Batis maritima-*dominated (Bama), and ponds or standing water (Pond). Exp is the experimental sediment application area/site. MAT is months after treatment.

**Fig 9:**
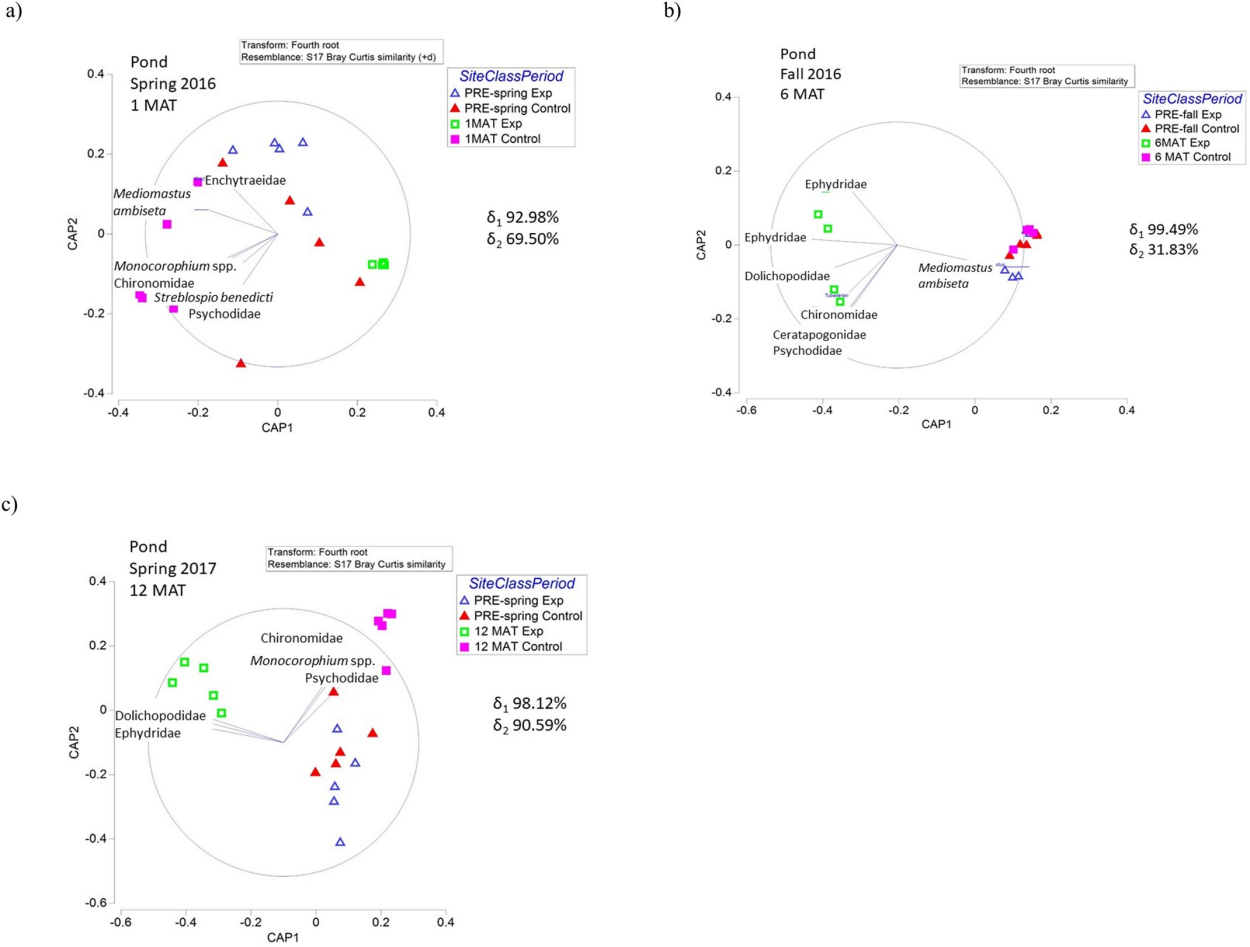
Infaunal invertebrate CAP plots showing the change in community composition of invertebrates for each sampling season (a,b,c) in Pond habitats compared to pre-sediment application data. Canonical correlation values (δ) for each axis are reported to show strength of the association between the multivariate data cloud and the hypothesized group differences. Habitats are abbreviated as follows: *Spartina foliosa*-dominated (Spfo), *Batis maritima-*dominated (Bama), and ponds or standing water (Pond). Exp is the experimental sediment application area/site. MAT is months after treatment.

The variation within the community composition of infaunal invertebrate species through time is explained by the CAP broken down by habitat. At 1 MAT in Spfo and Pond habitats, the post-sediment application experimental sites are separated from pre-sediment application and all control sites by a lack of invertebrates present at the experimental sites (especially Enchytraeidae, *Mediomastus californiensis*, *Monocorophium* spp.) (Figs 7A and 9A). The differences in the post-sediment application experimental sites for Bama habitats are also driven by a presence of Ephydridae larvae and also a lack of other invertebrates (Fig 8). The post-sediment application experimental sites at 6 MAT are separated by the presence of Ephydridae larvae and the absence of other invertebrate species in all habitat types (Figs 7B, 8B and 9B). The differences in the post-sediment application experimental sites in Pond habitats are also driven by the presence of several other species of insect larvae (Dolichopodidae, Chironomidae, Certatopogonidae) (Fig 9). Finally, similar to patterns seen at 1 MAT and 6 MAT, the community composition of the post-sediment application site at 12 MAT differed from the control site due to overall low abundances of invertebrates and due to the presence of only insect larvae (Figs 7C, 8C and 9C).

## Discussion

In the short-term (12 months), the addition of a deep sediment layer had dramatic negative effect on both vegetation and invertebrate communities in this marsh ecosystem. The lack of vegetation at 12 months’ post-sediment application as well as the significant decline in invertebrate abundance and alteration of the invertebrate community composition is likely due to a combination of interrelated factors and processes: the thickness of the sediment applied, the resulting supratidal elevation, harsh abiotic parameters, dispersal inhibition, and the characteristics of the dredge material. Given the relationship between plant cover and sediment-dwelling organisms in tidal marshes, we hypothesize that the slow recovery of invertebrates was related to the lack of plant cover (e.g. [51]).

### Thickness of sediment applied

Sediment addition experiments in marshes have been used to raise elevations and restore plant communities. Most of the ecosystems studied for this work were in the deltaic microtidal marshes of Louisiana, USA [17–19,52–55] and barrier marshes in North Carolina, USA [21], with a goal of sediment addition for restoration due to large-scale disturbances, such as marsh dieback, drought or regional subsidence. These studies used sediment depths ranging from 2–10 cm and found that, within a short timeframe (averaging one year), plant cover, plant productivity, aboveground biomass, and soil mineral matter all quickly increased following sediment application. Additional studies that monitored change over seven years indicated similar improvements in deltaic marshes post-application [20], and fourteen years in salt marsh islands of New York, USA [11], indicating that time scale of recovery is variable even within similar marsh types.

Overall, thinner sediment applications may damage plants but are unlikely to smother existing plant communities while thicker sediment applications seem to smother and significantly impact plant communities, resulting in slower vegetation recovery due to a need for plants to recolonize. Specifically, in a submerging coastal marsh in the Mississippi River Delta region of coastal Louisiana, USA, Ford et al. [19] found that a thin-layer sediment addition (2.3 cm) knocked down plants initially but the plant community recovered after 12 months. Thicker applications (e.g. 10–38 cm of sediment) studied by Cahoon and Cowan [54] smothered and killed most vegetation with recovery via recolonization just starting at 14 months after the sediment application. Thus, if restoration designs call for deeper sediment addition, slower plant community recovery is expected without active restoration strategies like replanting (as discussed later). Previous studies have shown the impacts of adding thin-layer sediment slurry on marsh plant communities, but more research is needed to investigate thicker sediment applications, such as those used in this study (32 cm). Non-deltaic, rapidly submerging marshes likely require deeper depths of applied sediment to prevent SLR submergence.

The recommended application depth will vary with management concerns, predicted SLR, and existing marsh elevations. It has generally been postulated that sediment additions that are less than 15 cm can be recolonized by re-sprouting of existing marsh vegetation, while additions that are greater than 15 cm are recolonized by new plants colonizing the area [56]. In our study with a sediment application depth of 32 cm, there is no evidence that the same plants that existed pre-sediment application grew through the dredge sediment [19,21]. This depth of sediment application combined with resultant harsh abiotic properties presumably prevented the re-emergence of existing vegetation and slowed recovery.

### Supratidal elevation

Similarly, prior studies conducted in deltaic microtidal salt marshes rarely raised the marsh platform above the tidal range. One such study, Stagg and Mendelssohn (2011), found that salt marshes subsidized with sediment to elevations 2–11 cm above MSL in deltaic marshes of coastal Louisiana, USA experienced less flooding stress, but sediment addition greater than 11 cm above MSL led to decreased platform stability [52].

In our study, sediment application resulted in a reduced inundation depth and less frequent inundation regime whereas the control site experienced a greater percentage of time under water in the same year [39]. Pre-sediment application, the experimental and control sites were inundated at most tide heights greater than approximately 0.46 m and 0.57 m above MSL, respectively, which was calculated to be 5.2 and 7.9% of the time [39]. Post-sediment application, the control site was inundated by tides greater than 0.36 m, which was 9.2% of the time while the experimental site was inundated only by tides higher than 0.48 m, which was calculated to be 2.3% of the time [39]. The sediment application raised the marsh elevation from intertidal to supratidal, which likely contributed to the slow establishment of the plants and invertebrate communities when compared to other short term studies [39]. Supratidal marshes occur above the intertidal zone and don’t receive daily tidal inundation but are flooded only during spring high tides and other high water events which can directly influences abiotic factors and the type of plants and invertebrates that develop [50]. While increasing marsh platform elevations was the ultimate purpose for this project and may be necessary to prevent submergence due to SLR, the short-term ecological implications of such a substantial elevation change had several negative outcomes for vegetation and invertebrate recovery.

### Colonization

This study was undertaken knowing that post-sediment application elevations may place the marsh surface at supratidal elevations. Supratidal elevations can limit colonization of vegetation and invertebrates. For plants the literature demonstrates that recovery is governed by optimal inundation and hydroperiod, high organic matter content concurrent with appropriate elevation, and rhizome survivability following burial (e.g. [50,57,58]). *Spartina foliosa* is found at the SBNWR at elevations up to 0.71 m above MSL [39]. Post-sediment application elevations range from 0.86 below MSL to 1.27 m above MSL [39], and it is therefore possible that certain areas on the sediment application site may not be suitable for the growth of *Spartina foliosa* and are above the optimal elevation range from some studies (e.g. [9]) surface vegetation. Although there is no evidence that within one year of sediment application *Spartina foliosa* or other high marsh plants colonizing the sediment application site, at 6 MAT newly colonized *Salicornia bigelovii* was just beginning to colonize throughout the site. This annual succulent species is an opportunistic colonizer that has a broad ecological tolerance and was not characteristic of the plant community before sediment application took place [59]. Long-lived perennials that are more reflective of the pre-sediment application community (*Salicornia pacifica*, *Spartina foliosa*, and *Batis maritima)* may take longer to return to the site and persist because seed delivery may be limited by the decreased flooding regime.

The rate at which invertebrates colonize an area is also influenced by the hydroperiod [57]. Restored marshes at higher elevations were found to have significantly fewer invertebrates than those at lower elevations when the elevation differed by 20 cm [60]. The invertebrates that were present are predominantly the larvae and pupae of mobile, algal-feeding insects, which are frequently early colonizers in marshes sites (e.g. [43–45,50,51]). The most abundant invertebrates (oligochaetes and polychaetes) found in the control site were almost entirely absent from the sediment application site. Several species of polychaetes exhibit a planktonic larval stage and may have been unable to recruit due to the decreased tidal inundation at the experiment site [57]. Delivery of both oligochaete and polychaete juveniles via sea grass rafts and algal mats has also been described as a mechanism for larval dispersal [50]. Rafted plant material was not encountered on the sediment application site, suggesting that elevation may also be limiting delivery of invertebrates by this mechanism. Once tidal inundation periods become longer and more frequent on the sediment application site due to further subsidence and SLR, opportunistic species like members of Capitellidae will likely begin to increase in abundance due to increased larval recruitment [61].

The initial decline in invertebrate abundance following sediment application was likely due not only to the smothering of existing communities and harsh abiotic environment conditions [19], but also the slow recovery of plants as trophic support and refuge to the site. Decaying plant matter serves as refugia for oligochaetes, and their root mats loosen the soil making it easier for oligochaetes to burrow, thus the absence of plants diminishes suitable habitat [60]. Over time, as vegetation develops in the marsh the invertebrate community generally begins to reflect communities more comparable to natural marshes. In restored *Spartina foliosa* marshes, it can take eight to ten years for the development of infaunal communities that resemble those of natural marshes [45,62].

### Changes in abiotic factors

Further limiting the colonization and influencing the community structure of plants and invertebrates is the high porewater salinity and temperature throughout the sediment application site. While we did not measure evaporation directly, the infrequent inundation combined with increased light intensity and temperature due to the absence of plants presumably resulted in increased evaporation (Fig 3 and S3 Table). Prior studies in northeastern USA [63], southern California, USA [51,64], Georgia in the southern USA [65], and in a coastal lagoon in central Argentina [66] have demonstrated that in the absence of shading, higher soil temperature, increased porewater salinities, and lower sediment pore water content can result and are most likely due to the increased sun exposure and subsequent evaporation. Lack of plant cover has been shown to impact infauna communities by altering the light and evaporation regime, decreasing species richness, increasing the proportion of insects, and decreasing the proportion of oligochaetes and polychaetes [51].

In addition to sediment application depth, the composition of the dredge material in this study may also have contributed to the lack of short-term recovery. Substrate conditions, both physical (e.g. grain size) and biochemical (e.g. redox potential), directly affect the growth and colonization of plants and invertebrates in marshes [57,67,68] and as reviewed in [62]. The dredged material used in this study was coarse-grained and low in organic matter. Initial increases in bulk density, indicating higher percentage sand, have been observed in previous sediment application studies (e.g. [19]). In this study, however, at 12 MAT, soil bulk density and percent organic matter had returned to or exceeded levels measured prior to sediment application despite percent silt and clay content remaining low (Fig 2). The discrepancies in sediment parameters between the control and post-sediment application in our study was greater than other studies and likely contributed to slower recovery of other parameters that we observed. Coarse-grained sediments drain water quickly creating aerobic conditions that lead to high rates of decomposition and low organic matter content [57]. Since organic matter is the main source of soil nutrients used for plant growth and serves as a major food source for invertebrates, coarse-grained sediment with low organic matter does not provide conditions conducive for salt marsh plants and invertebrates. Additionally, the faster drainage of coarse sediments leads to higher evaporation rates which contributed to the increase in salinity in this study (Fig 3 and S3 Table). These properties of sandy, salty sediment on the sediment application site are likely contributing to the delayed establishment of vegetation and consequently invertebrates in this marsh. A previous study of coarse-grained sandy sediment application to a predominantly sandy-mud back barrier North Carolina, USA marsh found that after two years, new plants were able to establish and grow to reflect pre-sediment application parameters after silty sediments were incorporated into the augmented sediment through natural marsh processes [21]. Over time, through bioturbation and sedimentation processes, natural sediments with higher silt and clay contents are expected to accumulate throughout the site, leading to increased nutrient levels and organic matter content which provide more favorable conditions for plants to continue to grow throughout the site.

### Future work and lessons learned

The overarching goal of this project was to build elevation capital to combat SLR submergence by using a thicker sediment addition method, and we will continue monitoring for additional years to monitor recovery. Given the relationships between sediment application depth and the delayed plant and invertebrate recovery (e.g. [17,52]), a thinner application of sediment may have expedited recovery; however, then the resultant elevation gain may not have been sufficient to combat SLR submergence for this site. Adjusting monitoring expectations to reflect a longer time frame for recovery may be important when assessing trade-offs in these high risk urbanized estuaries with limited sediment supply. Longer-term monitoring programs to fully understand recovery trajectories are important to invest in and implement for deep sediment application projects.

Adaptive management strategies to accelerate plant and invertebrate recovery time may be an important approach if the community does not recover at the pace expected or needed. There is evidence suggesting that the succession of a restored marsh relies on the overall amount of plant cover, so developing planting strategies for the site may help speed up recovery [42,57]. This has also been shown to be effective for long-term recovery following other thin-layer sediment addition projects [69]. Implementing a planting strategy using polyculture plots in salt marshes has been shown to increase plant cover by 80–100% in one year and increase species richness and canopy complexity [70]. Soil amendments such as nitrogen fertilization have been suggested for enhancing plant growth; however, it was found that despite immediately stimulating plant growth, nitrogen is not retained in the system and enhanced growth is not sustained once fertilization ends [71]. Rather, amending the substrate by importing finer marsh soils that incorporates rhizomes, roots, and microorganisms to the site is a preferred method to enhance plant establishment and growth [57,58]. One or more of these active restoration strategies can be considered as an important adaptive management restoration tool to achieve management goals and speed functional recovery following sediment application and will be considered for this project.

## Conclusions

Sediment addition projects are a unique approach to restoring and building marsh elevations. The impacts from these projects will vary depending on initial marsh bio-geomorphic properties, the depth of application, and the composition of sediment used. Considering our measured negative effects of this deeper sediment addition on vegetation and invertebrate communities in the first twelve months, continued monitoring and the identification of adaptive management strategies is important. Deeper sediment application projects on the Pacific Coast, USA and other areas may be necessary especially for those marshes located in urban estuaries where SLR adaptation strategies are limited.

Urbanized estuaries with marshes around the world are at risk of submergence from SLR due to sediment starvation, human development, and the interaction of these complex processes [27,72]. Without management actions, many of these marshes may be converted to mudflat and subtidal habitats as SLR rates outpace accretion (e.g. [27]). This study provided a unique, large-scale opportunity to explore the short-term impacts of sediment addition on important ecosystem features and processes. We measured a relatively slow trajectory of site recovery when compared with other regional systems and thinner application methods. Understanding that the recovery of the marsh is not occurring within 12 months is crucial for planning for effective restoration and monitoring programs in the future. Also, studying the effects of sediment application on a range of ecological settings will allow for a more complete understanding of its utilization as a management strategy for wetlands threatened by SLR. Evaluation of management strategies such as sediment application at various time scales with thorough pre- and post-monitoring is important to help design resilience strategies in the region and for subsiding wetlands in urban regions moving forward.

## Supporting information

S1 Table Pre- and post-application elevations at the sediment addition site. Sediment addition sampling locations (longitude, latitude), pre- and post-application elevations, and elevation change from pre- to post-sediment application.
(DOCX)

S2 Table Pre-application elevations at the control site. Control site sampling locations (longitude, latitude), pre-application elevations. Note: Post-application elevations at the control site were the same as pre-application elevations within the error of the DEM.
(DOCX)

S3 Table Pre-augmentation salinity compared to post-augmentation within sampling season by permutational ANOVAS. Bolded font indicates significant p-values. Habitats are abbreviated as follows: *Spartina foliosa*-dominated (Spfo), *Batis maritima-*dominated (Bama), and ponds or standing water (Pond). Pmc is the test statistic for the permutational ANOVAS using monte-carlo routines. MAT is months after treatment.
(DOCX)

S4 Table Plant parameters 1 MAT. Pre-Augmentation Data (Spring 2015) Compared to 1 Month Post-Augmentation (Spring 2016) by Two-Way ANOVAS or permutational ANOVAS for Plant Parameters. Bolded font indicates significant p-values. Habitats are abbreviated as follows: *Spartina foliosa*-dominated (Spfo), *Batis maritima-*dominated (Bama), and ponds or standing water (Pond). Pmc is the test statistic for the permutational ANOVAS using monte-carlo routines. MAT is months after treatment.
(DOCX)

S5 Table Plant parameters 6 MAT. Pre-Augmentation Data (Fall 2015) Compared to 6 Months Post-Augmentation (Fall 2016) by Two-Way ANOVAS or permutational ANOVAS for Plant Parameters. Bolded font indicates significant p-values. Habitats are abbreviated as follows: *Spartina foliosa*-dominated (Spfo), *Batis maritima-*dominated (Bama), and ponds or standing water (Pond). Pmc is the test statistic for the permutational ANOVAS using monte-carlo routines. MAT is months after treatment.
(DOCX)

S6 Table Plant parameters 12 MAT. Pre-Augmentation Data (Spring 2015) Compared to 12 Months Post-Augmentation (Spring 2017) by Two-Way ANOVAS or permutational ANOVAS for Plant Parameters. Bolded font indicates significant p-values. Habitats are abbreviated as follows: *Spartina foliosa*-dominated (Spfo), *Batis maritima-*dominated (Bama), and ponds or standing water (Pond). Pmc is the test statistic for the permutational ANOVAS using monte-carlo routines. MAT is months after treatment.
(DOCX)

S7 Table Pre-augmentation epifauna abundance compared to post-augmentation abundance within sampling season by permutational ANOVAS. Bolded font indicates significant p-values. Habitats are abbreviated as follows: *Spartina foliosa*-dominated (Spfo), *Batis maritima-*dominated (Bama), and ponds or standing water (Pond). Pmc is the test statistic for the permutational ANOVAS using monte-carlo routines. MAT is months after treatment.
(DOCX)

S8 Table Infauna parameters 1 MAT. Pre-Augmentation Data (Spring 2015) Compared to 1 Month Post-Augmentation (Spring 2016) by Two-Way ANOVAS or permutational ANOVAS for Infaunal Parameters. Bolded font indicates significant p-values. Habitats are abbreviated as follows: *Spartina foliosa*-dominated (Spfo), *Batis maritima-*dominated (Bama), and ponds or standing water (Pond). Pmc is the test statistic for the permutational ANOVAS using monte-carlo routines. MAT is months after treatment.
(DOCX)

S9 Table Infauna parameters 6 MAT. Pre-Augmentation Data (Fall 2015) Compared to 6 Months Post-Augmentation (Fall 2016) by Two-Way ANOVAS or permutational ANOVAS for Infaunal Parameters. Bolded font indicates significant p-values. Habitats are abbreviated as follows: *Spartina foliosa*-dominated (Spfo), *Batis maritima-*dominated (Bama), and ponds or standing water (Pond). Pmc is the test statistic for the permutational ANOVAS using monte-carlo routines. MAT is months after treatment.
(DOCX)

S10 Table Infauna parameters 12 MAT. Pre-Augmentation Data (Spring 2015) Compared to 12 Months Post-Augmentation (Spring 2017) by Two-Way ANOVAS or permutational ANOVAS for Infaunal Parameters. Bolded font indicates significant p-values. Habitats are abbreviated as follows: *Spartina foliosa*-dominated (Spfo), *Batis maritima-*dominated (Bama), and ponds or standing water (Pond). Pmc is the test statistic for the permutational ANOVAS using monte-carlo routines. MAT is months after treatment.
(DOCX)
