# Supplementary material for: Short-term impact of sediment addition on plants and invertebrates in a southern California salt marsh
Source: PLoS One. 2020 Nov 5;15(11):e0240597. doi: 10.1371/journal.pone.0240597 (PMC7644084; doi:10.1371/journal.pone.0240597)
Supplement: S1 Table — Sediment addition sampling locations (longitude, latitude), pre- and post-application elevations, and elevation change from pre- to post-sediment application. (DOCX) [file pone.0240597.s001.docx]

**S1 TABLE**. Sediment addition sampling locations (longitude, latitude), pre- and post-application elevations, and elevation change from pre- to post-sediment application.

| **Sampling Point** | **Latitude** | **Longitude** | **Pre-sediment application elevation (m, NAVD88)** | **Post-sediment application elevation (m, NAVD88)** | **Elevation change (m)** |
| --- | --- | --- | --- | --- | --- |
| 192 | 33.736834 | -118.078221 | 1.42 | 1.67 | 0.25 |
| 193 | 33.736982 | -118.07799 | 1.42 | 1.67 | 0.25 |
| 194 | 33.737028 | -118.077725 | 1.20 | 1.63 | 0.43 |
| 195 | 33.737075 | -118.077746 | 1.21 | 1.56 | 0.34 |
| 196 | 33.736756 | -118.07766 | 1.39 | 1.69 | 0.30 |
| 197 | 33.736515 | -118.077456 | 1.28 | 1.56 | 0.29 |
| 198 | 33.736676 | -118.077381 | 1.21 | 1.05 | -0.16 |
| 199 | 33.736595 | -118.077139 | 1.04 | null | null |
| 200 | 33.73652 | -118.078478 | 1.29 | 1.60 | 0.32 |
| 201 | 33.736297 | -118.078314 | 1.37 | 1.61 | 0.24 |
| 202 | 33.736143 | -118.078686 | 1.37 | 1.67 | 0.29 |
| 203 | 33.736029 | -118.07859 | 1.23 | 1.62 | 0.39 |
| 204 | 33.736304 | -118.07878 | 1.25 | 1.53 | 0.28 |
| 205 | 33.736115 | -118.078922 | 1.33 | 1.63 | 0.31 |
| 206 | 33.736 | -118.079126 | 1.22 | 1.64 | 0.42 |
| 207 | 33.735808 | -118.079143 | 1.31 | 1.92 | 0.62 |
| 208 | 33.735907 | -118.079278 | 1.34 | 1.73 | 0.39 |
| 209 | 33.735763 | -118.079521 | 1.27 | 1.61 | 0.34 |
| 210 | 33.735932 | -118.079134 | 1.34 | 1.72 | 0.38 |
| 211 | 33.735878 | -118.079037 | 1.33 | 1.68 | 0.35 |
| 212 | 33.735992 | -118.078597 | 1.18 | 1.57 | 0.39 |
| 213 | 33.735953 | -118.078409 | 1.30 | 1.64 | 0.34 |
| 214 | 33.736134 | -118.078238 | 1.33 | 1.64 | 0.30 |
