# Supplementary material for: Short-term impact of sediment addition on plants and invertebrates in a southern California salt marsh
Source: PLoS One. 2020 Nov 5;15(11):e0240597. doi: 10.1371/journal.pone.0240597 (PMC7644084; doi:10.1371/journal.pone.0240597)
Supplement: S2 Table — Control site sampling locations (longitude, latitude), pre-application elevations. Note: Post-application elevations at the control site were the same as pre-application elevations within the error of the DEM. (DOCX) [file pone.0240597.s002.docx]

**S2 TABLE.** Control site sampling locations (longitude, latitude), pre-application elevations. Note: Post-application elevations at the control site were the same as pre-application elevations within the error of the DEM.

| **Sampling Point** | **Latitude** | **Longitude** | **Elevation (m, NAVD88)** |
| --- | --- | --- | --- |
| 247 | 33.73495 | -118.083947 | 1.36 |
| 248 | 33.734901 | -118.083812 | 1.32 |
| 249 | 33.734855 | -118.08367 | 1.24 |
| 250 | 33.735021 | -118.083586 | 1.30 |
| 251 | 33.735022 | -118.083378 | 1.30 |
| 252 | 33.735346 | -118.082921 | 1.25 |
| 253 | 33.735541 | -118.082627 | 1.25 |
| 254 | 33.735524 | -118.082392 | 1.35 |
| 255 | 33.735689 | -118.082411 | 1.22 |
| 256 | 33.735371 | -118.082505 | 1.29 |
| 257 | 33.735125 | -118.082773 | 1.23 |
| 258 | 33.735026 | -118.082893 | 1.21 |
| 259 | 33.734841 | -118.083148 | 1.19 |
| 260 | 33.734781 | -118.083282 | 1.20 |
| 261 | 33.734586 | -118.083366 | 1.31 |
