# Supplementary material for: Short-term impact of sediment addition on plants and invertebrates in a southern California salt marsh
Source: PLoS One. 2020 Nov 5;15(11):e0240597. doi: 10.1371/journal.pone.0240597 (PMC7644084; doi:10.1371/journal.pone.0240597)
Supplement: S3 Table — Bolded font indicates significant p-values. Habitats are abbreviated as follows: Spartina foliosa-dominated (Spfo), Batis maritima-dominated (Bama), and ponds or standing water (Pond). Pmc is the test statistic for the permutational ANOVAS using monte-carlo routines. MAT is months after treatment. (DOCX) [file pone.0240597.s003.docx]

**S3 TABLE.** Pre-Augmentation Salinity Compared to Post-Augmentation Within Sampling Season by permutational ANOVAS

| Parameter/Sampling period | Habitat | SiteClass*Period^a^ | Result | Biological Interpretation |
| --- | --- | --- | --- | --- |
| Salinity  (1MAT, spring 2016) | Spfo  Bama  Pond | (pmc=0.235, pseudo F =1.53)  (**pmc=0.002**, pseudo F =14.069)  (pmc=0.389, pseudo F =0.81) | S15=S16  S15<S16  S15=S16 | No augmentation impact  Augmentation ↑ salinity  No augmentation impact |
| Salinity  (6 MAT, fall 2016) | Spfo  Bama  Pond | **(pmc=0.001**, pseudo F=15.81)  **(pmc=0.001**, pseudo F =25.49)  **(pmc=0.005**, pseudo F =9.30) | S15<S16  S15<S16  S15<S16 | Augmentation ↑ salinity  Augmentation ↑ salinity  Augmentation ↑ salinity |
| Salinity  (12 MAT, spring 2017) | Spfo  Bama  Pond | **(pmc=0.001**, pseudo F=58.99)  **(pmc=0.001**, pseudo F=22.33)  **(pmc=0.001**, pseudo F=114.22) | S15<S16  S15<S16  S15<S16 | Augmentation ↑ salinity  Augmentation ↑ salinity  Augmentation ↑ salinity |

Bolded font indicates significant p-values. Habitats are abbreviated as follows: *Spartina foliosa*-dominated (Spfo), *Batis maritima-*dominated (Bama), and ponds or standing water (Pond). Pmc is the test statistic for the permutational ANOVAS using monte-carlo routines. MAT is months after treatment.

^a^The interaction term represents the SiteClass (control vs impact) vs Period (before vs after impact) interaction, and a significant value is demonstration of an impact from thin-layer sediment addition.
