# Supplementary material for: Short-term impact of sediment addition on plants and invertebrates in a southern California salt marsh
Source: PLoS One. 2020 Nov 5;15(11):e0240597. doi: 10.1371/journal.pone.0240597 (PMC7644084; doi:10.1371/journal.pone.0240597)
Supplement: S5 Table — Pre-Augmentation Data (Fall 2015) Compared to 6 Months Post-Augmentation (Fall 2016) by Two-Way ANOVAS or permutational ANOVAS for Plant Parameters. Bolded font indicates significant p-values. Habitats are abbreviated as follows: Spartina foliosa-dominated (Spfo), Batis maritima-dominated (Bama), and ponds or standing water (Pond). Pmc is the test statistic for the permutational ANOVAS using monte-carlo routines. MAT is months after treatment. (DOCX) [file pone.0240597.s005.docx]

**S5 TABLE.** Plant Parameters 6 MAT. Pre-Augmentation Data (Fall 2015) Compared to 6 Months Post-Augmentation (Fall 2016) by Two-Way ANOVAS or permutational ANOVAS for Plant Parameters.

| Parameter | Habitat | SiteClass*Period^a^ | Result | Biological Interpretation |
| --- | --- | --- | --- | --- |
| Total Cover | Spfo  Bama  Pond | **(pmc=0.001**, pseudo F=58.19)  **(pmc=0.001**, pseudo F=45.48)  **(pmc=0.001**, pseudo F=17.29) | F15>F16  F15>F16  F15>F16 | Augmentation ↓ plant cover  Augmentation ↓ plant cover  Augmentation ↓ plant cover |
| Richness (S) | Spfo  Bama  Pond | (**pmc=0.001**, pseudo F=410.79)  (**pmc=0.001**, pseudo F=32.09)  (**pmc=0.044**, pseudo F=6.69) | F15>F16  F15>F16  F15>F16 | Augmentation ↓ richness  Augmentation ↓ richness  Augmentation ↓ richness |
| Diversity (H’) | Spfo  Bama  Pond | (**p=0.019**, F=6.79)  (**p<0.001**, F=301.92)  (**p<0.001**, F=83.17) | F15>F16  F15>F16  F15>F16 | Augmentation ↓ diversity  Augmentation ↓ diversity  Augmentation ↓ diversity |
| Evenness (J’) | Spfo  Bama  Pond | (**p=0.024**, F=6.24)  (**p<0.001**, F=190.76)  (**p<0.001**, F=148.01) | F15>F16  F15>F16  F15>F16 | Augmentation ↓ evenness  Augmentation ↓ evenness  Augmentation ↓ evenness |
| Community Composition | Spfo  Bama  Pond | (**pmc=0.001**, pseudo F=35.45)  (**pmc=0.002**, pseudo F=22.60)  (**pmc=0.001**, pseudo F=8.53) | F15≠F16  F15≠F16  F15≠F16 | Augmentation altered community  Augmentation altered community  Augmentation altered community |

Bolded font indicates significant p-values. Habitats are abbreviated as follows: *Spartina foliosa*-dominated (Spfo), *Batis maritima-*dominated (Bama), and ponds or standing water (Pond). Pmc is the test statistic for the permutational ANOVAS using monte-carlo routines. MAT is months after treatment.

^a^The interaction term represents the SiteClass (control vs impact) vs Period (before vs after impact) interaction, and a significant value is demonstration of an impact from thin-layer sediment addition.
