# Supplementary material for: Short-term impact of sediment addition on plants and invertebrates in a southern California salt marsh
Source: PLoS One. 2020 Nov 5;15(11):e0240597. doi: 10.1371/journal.pone.0240597 (PMC7644084; doi:10.1371/journal.pone.0240597)
Supplement: S6 Table — Pre-Augmentation Data (Spring 2015) Compared to 12 Months Post-Augmentation (Spring 2017) by Two-Way ANOVAS or permutational ANOVAS for Plant Parameters. Bolded font indicates significant p-values. Habitats are abbreviated as follows: Spartina foliosa-dominated (Spfo), Batis maritima-dominated (Bama), and ponds or standing water (Pond). Pmc is the test statistic for the permutational ANOVAS using monte-carlo routines. MAT is months after treatment. (DOCX) [file pone.0240597.s006.docx]

**S6 TABLE.** Plant Parameters 12 MAT. Pre-Augmentation Data (Spring 2015) Compared to 12 Months Post-Augmentation (Spring 2017) by Two-Way ANOVAS or permutational ANOVAS for Plant Parameters.

| Parameter | Habitat | SiteClass*Period^a^ | Result | Biological Interpretation |
| --- | --- | --- | --- | --- |
| Total Cover | Spfo  Bama  Pond | **(pmc=0.001**, pseudo F=72.96)  **(pmc=0.001**, pseudo F=27.94)  (pmc=0.068, pseudo F=3.45) | S15>S16  S15>S16  S15=S16 | Augmentation ↓ plant cover  Augmentation ↓ plant cover  No augmentation impact |
| Richness (S) | Spfo  Bama  Pond | (**pmc=0.001**, pseudo F=346.79)  (**pmc=0.003**, pseudo F=10.11)  (pmc=0.078, pseudo F=3.47) | S15>S16  S15>S16  S15>S16 | Augmentation ↓ richness  Augmentation ↓ richness  Augmentation ↓ richness |
| Diversity (H’) | Spfo  Bama  Pond | (**pmc=0.001**, pseudo F=15.93)  (**pmc=0.001**, pseudo F=29.42)  (**pmc=0.001**, pseudo F=1786.5) | S15>S16  S15>S16  S15>S16 | Augmentation ↓ diversity  Augmentation ↓ diversity  Augmentation ↓ diversity |
| Evenness (J’) | Spfo  Bama  Pond | (**pmc=0.002**, pseudo F=16.22)  (**pmc=0.001**, pseudo F=28.01)  (**pmc=0.001**, pseudo F=470.9) | S15>S16  S15>S16  S15>S16 | Augmentation ↓ evenness  Augmentation ↓ evenness  Augmentation ↓ evenness |
| Community Composition | Spfo  Bama  Pond | (**pmc=0.001**, pseudo F=30.42)  (**pmc=0.001**, pseudo F=13.78)  (**pmc=0.003**, pseudo F=12.28) | S15≠S17  S15≠S17  S15≠S17 | Augmentation altered community  Augmentation altered community  Augmentation altered community |

Bolded font indicates significant p-values. Habitats are abbreviated as follows: *Spartina foliosa*-dominated (Spfo), *Batis maritima-*dominated (Bama), and ponds or standing water (Pond). Pmc is the test statistic for the permutational ANOVAS using monte-carlo routines. MAT is months after treatment.

^a^The interaction term represents the SiteClass (control vs impact) vs Period (before vs after impact) interaction, and a significant value is demonstration of an impact from thin-layer sediment addition.
