# Supplementary material for: Short-term impact of sediment addition on plants and invertebrates in a southern California salt marsh
Source: PLoS One. 2020 Nov 5;15(11):e0240597. doi: 10.1371/journal.pone.0240597 (PMC7644084; doi:10.1371/journal.pone.0240597)
Supplement: S7 Table — Bolded font indicates significant p-values. Habitats are abbreviated as follows: Spartina foliosa-dominated (Spfo), Batis maritima-dominated (Bama), and ponds or standing water (Pond). Pmc is the test statistic for the permutational ANOVAS using monte-carlo routines. MAT is months after treatment. (DOCX) [file pone.0240597.s007.docx]

**S7 TABLE.** Pre-Augmentation Epifauna Abundance Compared to Post-Augmentation Abundance Within Sampling Season by permutational ANOVAS

| Parameter/Sampling period | Habitat | SiteClass*Period^a^ | Result | Biological Interpretation |
| --- | --- | --- | --- | --- |
| Abundance (N)  (1 MAT, spring 2016) | Spfo  Bama  Pond | (pmc=0.061, pseudo F=4.43)  (**pmc=0.003**, pseudo F=14.80)  (**pmc=0.002**, pseudo F=14.82) | S15=S16  S15>S16  S15>S16 | No augmentation impact  Augmentation ↓ abundance  Augmentation ↓ abundance |
| Abundance (N)  (6 MAT, fall 2016) | Spfo  Bama  Pond | (pmc=0.331, pseudo F=1.01)  (**pmc=0.002**, pseudo F=16.35)  (pmc=0.194, pseudo F=1.74) | F15=F16  F15>F16  F15=F16 | No augmentation impact  Augmentation ↓ abundance  No augmentation impact |
| Abundance (N)  (12 MAT, spring 2017) | Spfo  Bama  Pond | **(pmc=0.037,** pseudo F=4.91)  **(pmc=0.002**, pseudo F=14.14)  **(pmc=0.001**, pseudo F=65.77) | S15>S17  S15>S17  S15>S17 | Augmentation ↓ abundance  Augmentation ↓ abundance  Augmentation ↓ abundance |

Bolded font indicates significant p-values. Habitats are abbreviated as follows: *Spartina foliosa*-dominated (Spfo), *Batis maritima-*dominated (Bama), and ponds or standing water (Pond). Pmc is the test statistic for the permutational ANOVAS using monte-carlo routines. MAT is months after treatment.

^a^The interaction term represents the SiteClass (control vs impact) vs Period (before vs after impact) interaction, and a significant value is demonstration of an impact from thin-layer sediment addition.
