# Supplementary material for: Short-term impact of sediment addition on plants and invertebrates in a southern California salt marsh
Source: PLoS One. 2020 Nov 5;15(11):e0240597. doi: 10.1371/journal.pone.0240597 (PMC7644084; doi:10.1371/journal.pone.0240597)
Supplement: S8 Table — Pre-Augmentation Data (Spring 2015) Compared to 1 Month Post-Augmentation (Spring 2016) by Two-Way ANOVAS or permutational ANOVAS for Infaunal Parameters. Bolded font indicates significant p-values. Habitats are abbreviated as follows: Spartina foliosa-dominated (Spfo), Batis maritima-dominated (Bama), and ponds or standing water (Pond). Pmc is the test statistic for the permutational ANOVAS using monte-carlo routines. MAT is months after treatment. (DOCX) [file pone.0240597.s008.docx]

**S8 TABLE.** Infauna Parameters 1 MAT. Pre-Augmentation Data (Spring 2015) Compared to 1 Month Post-Augmentation (Spring 2016) by Two-Way ANOVAS or permutational ANOVAS for Infaunal Parameters

| Parameter | Habitat | SiteClass*Period^a^ | Result | Biological Interpretation |
| --- | --- | --- | --- | --- |
| Abundance (N) | Spfo  Bama  Pond | (**p<0.001**, F=20.38)  (**p<0.001**, F=28.25)  (**p=0.003**, F=11.67) | S15>S16  S15>S16  S15>S16 | Augmentation ↓ abundance  Augmentation ↓ abundance  Augmentation ↓ abundance |
| Richness (S) | Spfo  Bama  Pond | (**p<0.001**, F=26.23)  (**p<0.001**, F=54.11)  (**p<0.001**, F=29.79) | S15>S16  S15>S16  S15>S16 | Augmentation ↓ richness  Augmentation ↓ richness  Augmentation ↓ richness |
| Diversity (H’) | Spfo  Bama  Pond | (**p=0.003**, F=11.90)  (**p<0.001**, F=32.64)  (**p=0.002**, F=22.47) | S15>S16  S15>S16  S15>S16 | Augmentation ↓ diversity  Augmentation ↓ diversity  Augmentation ↓ diversity |
| Evenness (J’) | Spfo  Bama  Pond | (**pmc=0.032**, pseudo F=4.86)  **(pmc=0.001**, pseudo F=155.59)  **(pmc=0.002**, pseudo F=13.94) | S15>S16  S15>S16  S15>S16 | Augmentation ↓ evenness  Augmentation ↓ evenness  Augmentation ↓ evenness |
| Community Composition | Spfo  Bama  Pond | (**pmc=0.003**, F=5.15)  (**pmc=0.001**, F=15.09)  (**pmc=0.001**, F=7.67) | S15≠S16  S15≠S16  S15≠S16 | Augmentation altered community  Augmentation altered community  Augmentation altered community |

Bolded font indicates significant p-values. Habitats are abbreviated as follows: *Spartina foliosa*-dominated (Spfo), *Batis maritima-*dominated (Bama), and ponds or standing water (Pond). Pmc is the test statistic for the permutational ANOVAS using monte-carlo routines. MAT is months after treatment.

^a^The interaction term represents the SiteClass (control vs impact) vs Period (before vs after impact) interaction, and a significant value is demonstration of an impact from thin-layer sediment addition.
