# Supplementary material for: Short-term impact of sediment addition on plants and invertebrates in a southern California salt marsh
Source: PLoS One. 2020 Nov 5;15(11):e0240597. doi: 10.1371/journal.pone.0240597 (PMC7644084; doi:10.1371/journal.pone.0240597)
Supplement: S9 Table — Pre-Augmentation Data (Fall 2015) Compared to 6 Months Post-Augmentation (Fall 2016) by Two-Way ANOVAS or permutational ANOVAS for Infaunal Parameters. Bolded font indicates significant p-values. Habitats are abbreviated as follows: Spartina foliosa-dominated (Spfo), Batis maritima-dominated (Bama), and ponds or standing water (Pond). Pmc is the test statistic for the permutational ANOVAS using monte-carlo routines. MAT is months after treatment. (DOCX) [file pone.0240597.s009.docx]

**S9 TABLE.** Infauna Parameters 6 MAT. Pre-Augmentation Data (Fall 2015) Compared to 6 Months Post-Augmentation (Fall 2016) by Two-Way ANOVAS or permutational ANOVAS for Infaunal Parameters

| Parameter | Habitat | SiteClass*Period^a^ | Result | Biological Interpretation |
| --- | --- | --- | --- | --- |
| Abundance (N) | Spfo  Bama  Pond | (**p<0.001**, F=26.69)  (**p=0.006**, F=9.74)  (p=0.186, F=1.90) | F15>F16  F15>F16  F15=F16 | Augmentation ↓ abundance  Augmentation ↓ abundance  No augmentation impact |
| Richness (S) | Spfo  Bama  Pond | (**p<0.001**, F=14.07)  (p=0.050**,** F=4.55)  (p=0.119, F=2.71) | F15>F16  F15=F16  F15=F16 | Augmentation ↓ richness  No augmentation impact  No augmentation impact |
| Diversity (H’) | Spfo  Bama  Pond | (**p<0.001**, F=30.66)  (p=0.081, F=3.49)  (p=0.263, F=1.34) | F15>F16  F15=F16  F15=F16 | Augmentation ↓ diversity  No augmentation impact  No augmentation impact |
| Evenness (J’) | Spfo  Bama  Pond | **(pmc=0.0421**, pseudo F=6.844)  (pmc=0.355, pseudo F=0.958)  (pmc=0.358, pseudo F=0.757) | S15>S16  S15=S16  S15=S16 | Augmentation ↓ evenness  No augmentation impact  No augmentation impact |
| Community Composition | Spfo  Bama  Pond | (**pmc=0.003**, F=3.92)  (**pmc=0.001**, F=5.33)  (**pmc=0.001**, F=5.60) | F15≠F16  F15≠F16  F15≠F16 | Augmentation altered community  Augmentation altered community  Augmentation altered community |

Bolded font indicates significant p-values. Habitats are abbreviated as follows: *Spartina foliosa*-dominated (Spfo), *Batis maritima-*dominated (Bama), and ponds or standing water (Pond). Pmc is the test statistic for the permutational ANOVAS using monte-carlo routines. MAT is months after treatment.

^a^The interaction term represents the SiteClass (control vs impact) vs Period (before vs after impact) interaction, and a significant value is demonstration of an impact from thin-layer sediment addition.
