# Supplementary material for: Short-term impact of sediment addition on plants and invertebrates in a southern California salt marsh
Source: PLoS One. 2020 Nov 5;15(11):e0240597. doi: 10.1371/journal.pone.0240597 (PMC7644084; doi:10.1371/journal.pone.0240597)
Supplement: S10 Table — Pre-Augmentation Data (Spring 2015) Compared to 12 Months Post-Augmentation (Spring 2017) by Two-Way ANOVAS or permutational ANOVAS for Infaunal Parameters. Bolded font indicates significant p-values. Habitats are abbreviated as follows: Spartina foliosa-dominated (Spfo), Batis maritima-dominated (Bama), and ponds or standing water (Pond). Pmc is the test statistic for the permutational ANOVAS using monte-carlo routines. MAT is months after treatment. (DOCX) [file pone.0240597.s010.docx]

**S10 TABLE.** Infauna Parameters 12 MAT. Pre-Augmentation Data (Spring 2015) Compared to 12 Months Post-Augmentation (Spring 2017) by Two-Way ANOVAS or permutational ANOVAS for Infaunal Parameters

| Parameter | Habitat | SiteClass*Period^a^ | Result | Biological Interpretation |
| --- | --- | --- | --- | --- |
| Abundance (N) | Spfo  Bama  Pond | (**p<0.001**, F=26.69)  (**p=0.006**, F=9.93)  (p=0.135, F=2.49) | S15>S17  S15>S17  S15=S17 | Augmentation ↓ abundance  Augmentation ↓ abundance  No augmentation impact |
| Richness (S) | Spfo  Bama  Pond | (**p=0.001**, F=16.98)  (**p=0.005**, F=10.18)  (p=0.117, F=2.74) | S15>S17  S15>S17  S15=S17 | Augmentation ↓ richness  Augmentation ↓ richness  No augmentation impact |
| Diversity (H’) | Spfo  Bama  Pond | (**p=0.015**, F=2.25)  (**p=0.013**, F=7.91)  (p=0.146, F=2.33) | S15>S17  S15>S17  S15=S17 | Augmentation ↓ diversity  Augmentation ↓ diversity  No augmentation impact |
| Evenness (J’) | Spfo  Bama  Pond | (pmc=0.680, pseudo F=0.18)  **(pmc=0.044**, pseudo F=5.02)  (pmc=0.295, pseudo F=1.09) | S15=S17  S15>S17  S15>S17 | No augmentation impact Augmentation ↓ evenness  No augmentation impact |
| Community Composition | Spfo  Bama  Pond | (**pmc=0.008**, F=3.79)  (**pmc=0.001**, F=6.82)  (**pmc=0.008**, F=3.04) | F15≠F17  F15≠F17  F15≠F17 | Augmentation altered community  Augmentation altered community  Augmentation altered community |

Bolded font indicates significant p-values. Habitats are abbreviated as follows: *Spartina foliosa*-dominated (Spfo), *Batis maritima-*dominated (Bama), and ponds or standing water (Pond). Pmc is the test statistic for the permutational ANOVAS using monte-carlo routines. MAT is months after treatment.

^a^The interaction term represents the SiteClass (control vs impact) vs Period (before vs after impact) interaction, and a significant value is demonstration of an impact from thin-layer sediment addition.
